# Assimilation of Nanoparticles of SiC, ZrC, and WC with Polyaryletherketone for Performance Augmentation of Adhesives

**DOI:** 10.3390/nano13061028

**Published:** 2023-03-13

**Authors:** Umesh Marathe, Jayashree Bijwe

**Affiliations:** Centre for Automotive Research and Tribology (CART) (Formerly ITMMEC), Indian Institute of Technology, New Delhi 110016, India; umeshmarathe86@gmail.com

**Keywords:** adhesives, polyaryletherketone, thermal conductivity, lap shear strength, nanocomposites

## Abstract

The present paper reports the analyses of results obtained from experiments carried out to explore the challenge of homogeneous, uniform, and deagglomerated dispersion of ultra-heavy nanoparticles (NPs) in the high-performance polyaryletherketone (PAEK) matrix. An equal and fixed amount of (0.5 vol. %) NPs of silicon carbide (SiC), zirconium carbide (ZrC), and tungsten carbide (WC) were dispersed in a PAEK matrix and compression molded to develop three different nanocomposites. Simultaneously, nano-adhesives of the same composition were also developed to join the stainless steel adherends. The composites and adhesives were characterized for their physical, thermal, thermo-mechanical, thermal conductivity (TC), and lap shear strength (LSS) behavior. It was observed that SiC NPs performed significantly better than ZrC and WC NCs in all performance properties (LSS: 154%, TC: 263%, tensile strength: 21%). Thermal conductivity (TC) and tensile properties were validated using various predictive models, such as the rule of mixture parallel model, the Chiew and Glandt model, and the Lewis model. Scanning electron micrographs were used for the morphological analysis of LSS samples to detect macro- and micro-failure. Micrographs showed evidence of micro-striation and plastic deformation as a micromodel, as well as mixed failure, i.e., adhesive–cohesive as a macro-failure mode.

## 1. Introduction

Ever since their emergence as a potent soft material, polymeric nanocomposites have created their own domain of applications in automobiles, sports, space equipment/accessories, implants, food sensors, adhesives, and scores of other related areas. Polymer-based adhesives have an edge over conventional joining techniques such as welding and riveting since the latter methods are prone to corrosion (galvanic corrosion due to the usage of distinct components and environment), fretting, and fatigue due to the presence of mechanical tolerance and a lack of vibration-damping ability. Polymeric adhesives, especially nanocomposite (NC)-based adhesives, provide remedies without adversely affecting or compromising the performance properties. Most of the research papers and documents available in the literature on polymeric adhesives are focused on epoxy adhesives and composites filled with various carbonaceous nano-fillers [1,2,3,4,5,6]. For instance, due to the addition of 1 wt. % single-walled carbon nanotubes (SWCNTs) in the epoxy matrix, a 30% increase in peel strength has been reported [5]. However, it was accompanied by a 10–15% reduction in lap shear strength (LSS). Gültekin et al. [7] have reported ~20% improvement in LSS for epoxy filled with graphene (1 wt. %). Akpinar et al. [8] developed LSS joints using three different epoxy resins (flexible, tough, and rigid) and COOH functionalized CNTs, graphene, and fullerene (2 wt. % each) and reported a 276% improvement in LSS failure load due to the addition of graphene to rigid epoxy. However, improvement by fullerene and CNTs was marginal. For nano-adhesives (NAs) based on different epoxy resins, an improvement in stress by 32, 84, 44, and 140% with graphene, graphene oxide, CNTs, and fullerene respectively has been reported [8]. Similar studies have reported that the addition of NPs (such as fullerene and boron nitride nanotubes) improves the performance properties of epoxies, polyethylene, or polyvinyl alcohol (PVA) [9,10,11,12]. High-performance polymers such as polyaryletherketone (PAEK), polyetherketoneketone (PEKK), and polyetheretherketone (PEEK) are preferred over other polymeric matrices because of their high thermal stability, T_g_ (glass transition temperature), service temperature, mechanical properties, and their retention at elevated temperatures. PAEK is a comparatively newly commercialized polymer and has not been explored in depth for various applications, especially for nano-adhesives (NAs). Pascual et al. [13] have reported on their nanocomposites [NCs], and Panda et al. [14,15,16] have reported on the tribology of solid-lubricated NCs based on PAEK. Kadiyala et al. [17,18,19,20,21] have reported studying the effect of two types of ceramic particles, SiC (silicon carbide) and B_4_C (boron carbide) (nano- and micro-sized), in varying concentrations on the LSS using PAEK, PEEK, and polyethersulphone (PES) as matrices. Overall, SiC NPs in PAEK proved most beneficial, with an improvement of 116%. Boron carbide (20%) micro-particles showed improvements of 70% and 90% at room temperature and 300 °C, respectively. The particles of hard metal carbides act in a dual manner. They made scratches on the coupon surfaces due to their hard and sharp edges and strengthened the bulk by acting as nucleating reinforcement in the bulk region.

In the current study, the potential of NPs of silicon carbide (SiC), zirconium carbide (ZrC), and tungsten carbide (WC) in identical volume fractions in PAEK powder has been explored for possible performance enhancement as adhesives by developing NCs and nano-adhesives (NAs). The carbides were selected based on their high hardness (SiC = 2580 kg/mm^2^, ZrC = 2890 kg/mm^2^, WC = 2400 kg/mm^2^). Silicon carbide is a hard refractory material that exists in multiple crystalline structures. It is known for its high hardness, high thermal conductivity (TC) (120–270 W/mK), and low coefficient of thermal expansion with relatively low density, i.e., 3.21 g/cc. ZrC is a hard crystalline ceramic material from the interstitial metal carbide group with a cubic crystal structure. It has a low TC (20 W/mK) and a density of 6.73 g/cc. Tungsten carbide (WC) is a transition metal carbide with a metal-like appearance and very high density, i.e., 15.63 g/cc. WC has two forms, i.e., hexagonal and cubic high-temperature forms, with distinct structures.

## 2. Experimental

### 2.1. Materials

Polyaryletherketone (PAEK) is one of the least explored polymers for formulating NCs/NAs, mainly because of the processing challenges involved. PAEK was selected as a matrix material to develop NCs/NAs and was purchased from Gharda Chemicals Ltd. in Mumbai, India, under the trade name G-PAEK 1200P in a powdery form. Nanoparticles (NPs) of three types of carbides were selected as particulate reinforcement, and details are given in Table 1.

Coupons of stainless steel (grade 316) (15 mm (width) × 65 mm (length) × 1.5 mm (thickness)) with R_a_ of 0.15–0.20 µm were selected as adherends to develop the adhesive joints following ASTM D 1002.

### 2.2. Preparation of Nano-Mixtures

A probe sonicator (a ChromeTech ultrasonic processor with a probe diameter of 8–10 mm, 800 W power, cycle—the pulse of 3 s (on and off)) was employed to deagglomerate the NPs in an ethanol medium, followed by mixing them with PAEK powder. The measured weight of NPs was split into 5 parts, where each part was added to 100 mL of ethanol followed by 30 min of probe sonication. Simultaneously, an ethanol-PAEK suspension was prepared in 1500 mL of ethanol solvent. A nanosuspension of all five parts was added to this suspension after transferring it to a 2L beaker, followed by overnight magnetic stirring with slow heating. Post-magnetic stirring, the thick slurry was taken out and dried in the oven at 60 °C for 24 h. The dried powder of the nano-mixture was used to develop nanocomposites (NCs) and nano-adhesives (NAs). Table 2 lists the NCs/NAs compositions and their respective codes.

### 2.3. Development of Nanocomposite (NC) Sheets and LSS Joints

- NC sheets

**Figure 1:**
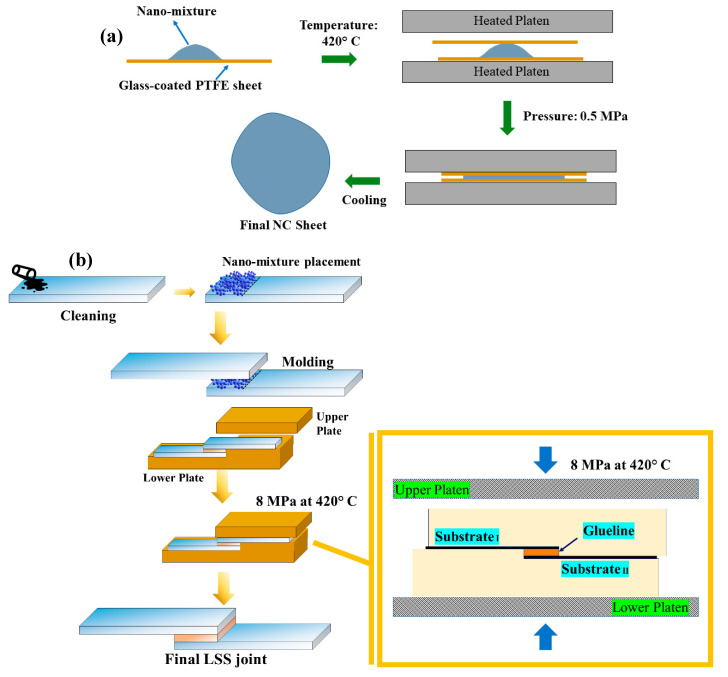
Schematics for (**a**) nanocomposite sheet fabrication and (**b**) the LSS joint preparation process.

The nano-mixtures, which dried at 70 °C, were used to develop the sheets of NCs by compression molding. PTFE-coated glass fabric sheets were used for ease in releasing the films once molding was completed. The nano-mixture (30–40 g) was placed between these sheets placed on a compression molding platen to obtain the film of approx. 200 mm × 200 mm × 0.50 mm dimensions. The platens were closed, and 0.5 MPa pressure was applied at 420 °C. The prepared sheet was then allowed to cool naturally and cut into pieces with the required dimensions for various characterizations. The schematic of the process is shown in Figure 1a.

- Lap shear joints

The lap shear strength (LSS) joints were developed as per ASTM D 1002. Figure 1b explains the process of placing the mixture and then molding a joint. SS coupons were cleaned with acetone twice to ensure degreasing and decontamination. Furthermore, one of the coupons was placed in the mold, and 0.10 g of the nano-mixture was spread evenly on the area of the joint (15 mm × 15 mm) on the first coupon, as shown in Figure 1b (schematics of preparation and molding), followed by the placement of another coupon, and finally closing the mold. The whole mold assembly was kept in a compression machine, and the temperature was allowed to reach 420 °C. A pressure of 8 MPa was then applied, followed by 5 breathing cycles. The joints were allowed to cool naturally to room temperature.

## 3. Characterization

### 3.1. Physical Properties

The physical properties of developed NCs include theoretical density, practical density, and void content. The theoretical density was calculated using the rule of mixture, whereas the practical density was calculated experimentally using ASTM D 792. Each sample was repeated at least eight times to ensure repeatability. Practical density was calculated using Equation (1), where W_air_, W_liquid_ and ρ_liquid_ are weight in air, weight in liquid and density of liquid respectively.

(1) $$\rho_{c}=\frac{\rho_{\mathrm{liquid}}\times {\;W}_{\mathrm{air}}}{W_{\mathrm{air}}-W_{\mathrm{liquid}}}$$

### 3.2. Dispersion by FE-SEM

FE-SEM (JEOL, Tokyo, Japan, JSM 7800F) was used to observe the dispersion of the NPs in the NC sheet. The NC sheets were fractured mechanically and observed under the FE-SEM.

### 3.3. Surface-Free Energy Measurements on NC Sheets

A few recent studies indicate that the nature of filler size affects the surface free energy (SFE) and contact angle. The contact angles were measured using a Krauss goniometer. The NC sheet was pasted on the mild steel surface and polished with a double-disc polishing machine with 1000–4000 grade polish paper. The finely polished surface of the NC sheet was used to measure the contact angle and subsequently the SFE using the Fowkes method. The contact angles were measured with deionized water (polar) as well as n-hexane (non-polar) with a drop volume of 2 µL (fitting method: ellipse). The measurements on each sample were repeated at least 10–12 times to ensure repeatability. The average value of the measured readings was considered for the calculations.

### 3.4. X-ray Diffraction Studies

X-ray diffraction (XRD) was used to understand polymer crystallinity and crystalline arrangements in NCs. The percentage crystallinity of the composites was calculated using the amorphous and crystalline regions observed in the diffractogram obtained with the help of Phillips XPERT-PRO, by Phillips, Slovak Republic (1.5418 Å (CuKα)) at 2Θ range of 10° to 80° and a scan rate of 5°/min.

### 3.5. Thermogravimetric Analysis (TGA)

TGA was used to understand the effect of loading different types of NPs into the PAEK matrix. Linseis 1000PT was used to understand the thermal stability of developed composites in the air (range 30–900 °C) using a 10 °C ramp rate. Linseis Evaluation Software (by Linseis, Selb, Germany) and Origin 2020 (by OrginLab, Northampton, MA, USA) were used to analyze data.

### 3.6. Thermal Conductivity

Thermal conductivity (TC) of composites was measured using TA Instruments DTC 300 thermal conductivity analyzers (guarded plate type), and sample area dimensions 1963.5 mm^2^ (for DTC 300) were used to measure the through-plain thermal conductivity.

### 3.7. Dynamic Mechanical Analysis

Dynamic mechanical analysis, i.e., DMA measurements, was conducted using TA instruments in tensile mode from 30 to 350 °C. The ramp rate was maintained at 10 °C/min.

### 3.8. Tensile Properties

The tensile strength and moduli were calculated with the help of a Shimadzu (Kyoto, Japan) universal testing machine (UTM). The measurements were taken at room temperature with a gauge length of 85 mm and a 15 mm grip on each side. The developed NC sheets were used to conduct the tensile strength experiment. The sheets were cut to the above-given dimensions and loaded with the help of tensile jaws housed in the UTM. The cross-head speed was maintained at 1.5 mm/min. Each sample was repeated six times to ensure repeatability.

### 3.9. Lap Shear Strength (LSS)

The LSS was used to analyze the strength of the developed lap shear joint. The tests were conducted as per ASTM D1002 in a tensile mode at 1.3 mm/min of cross-head speed on a Shimadzu UTM (load sensor of 10 kN), and the maximum load was noted. The LSS was calculated using Equation (2):

LSS = P/bL (2)

where P is the maximum load recorded, b is the width of the joint, and L is the overlap length. Five specimens were tested, and the average readings were reported as the LSS value for that sample.

### 3.10. Failure Analysis

After the failure of LSS joints, the samples were examined under the scanning electron microscope (EVO 10, Zeiss, Jena, Germany) to study the failure mechanisms.

## 4. Results and Discussion

### 4.1. Physical Properties

Table 2 depicts the densities of the NCs. The addition of heavier ceramic particles (SiC, ZrC, and WC) than PAEK led to an increase in the density of the composites. Despite remarkable variation in densities of particles (Table 2), densities of composites did not vary much because they were added as per vol. % and not as wt. %. Since the composites were processed using a compression molding machine, only 1–2 void % were noticed.

### 4.2. Dispersion by FE-SEM

**Figure 2:**
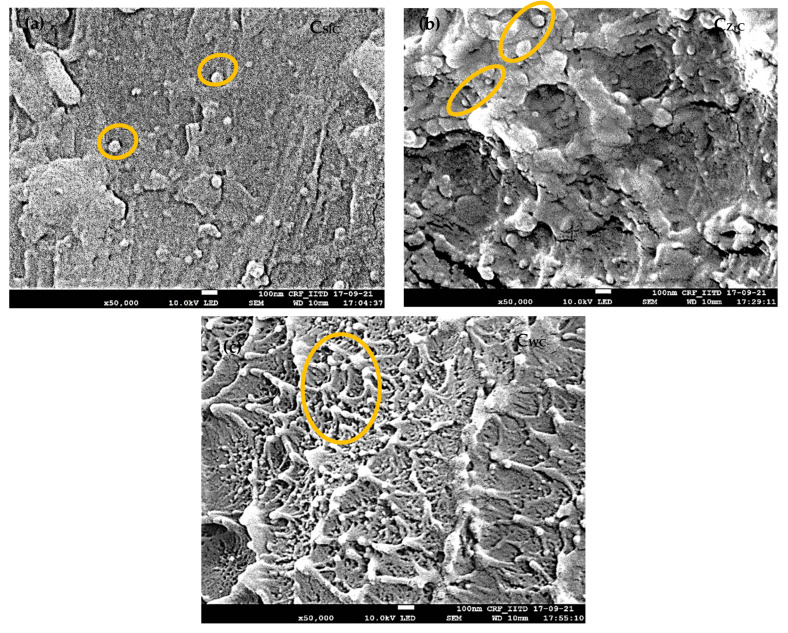
FE-SEM micrographs of films showing dispersion and range of size of NPs; (**a**) C_SiC_ 60–80 nm, (**b**) C_ZrC_ 70–90 nm, and (**c**) C_WC_ 50–70 nm.

Figure 2 shows the extent of dispersion for filled NPs in the PAEK. The samples were impact fractured and scanned under the FE-SEM to observe the extent of the dispersion and distribution of NPs.

All the NCs showed excellent dispersion and distribution of NPs. In further studies, it was observed that the extent of dispersion played a crucial role in controlling performance properties. Moreover, the NPS used in the study were high-density ceramic particles with a tendency for sedimentation. It was challenging to disperse them throughout the slurry of PAEK powder and medium slurry. It is evident from Figure 2 that the probe sonication coupled with mechanical stirring led to a non-sedimented, well-dispersed nano-mixture. Moreover, it is a kind of genesis for improved performance properties. Well-dispersed NPs throughout the bulk of the matrix act as nucleating agents and improve the crystallinity of the polymer, and the high surface area bestowed by NPs results in a high interfacial volume, leading to improved stress transfer and module [24].

### 4.3. Surface-Free Energy Measurements on NC Sheets

**Figure 3:**
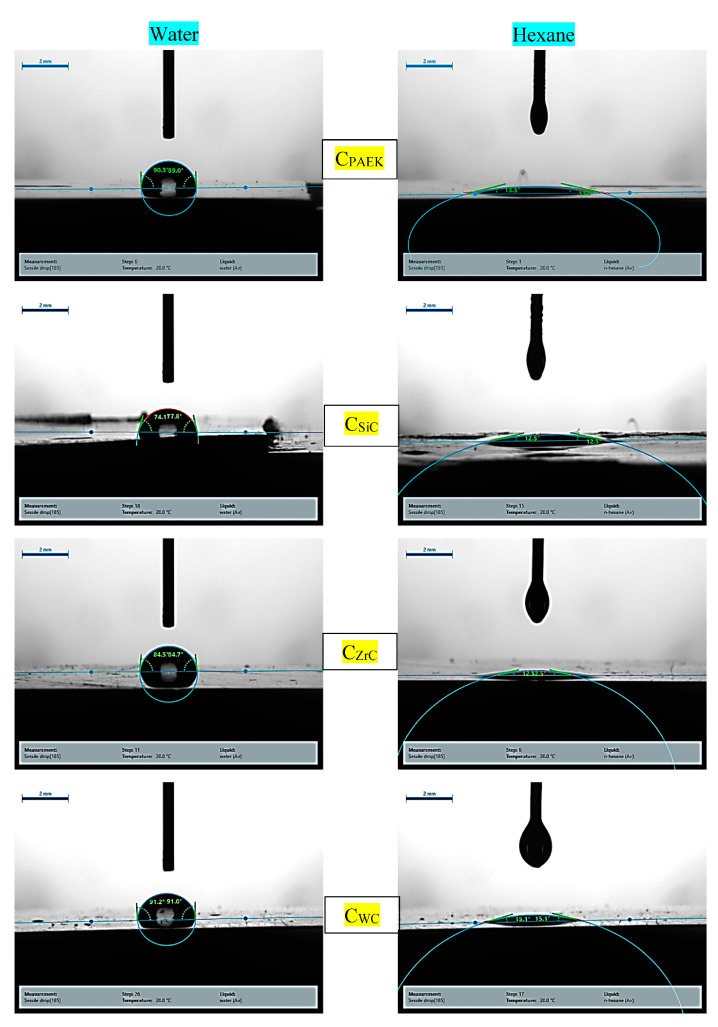
Contact angle images for developed NCs.

**Figure 4:**
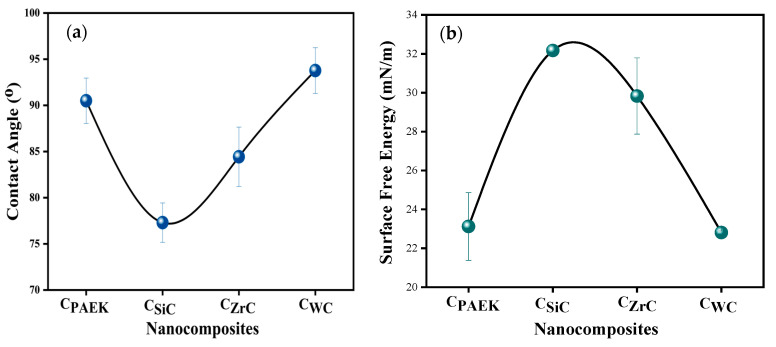
(**a**) Contact angles for NCs; (**b**) surface-free energy for NCs.

Figure 3 depicts the contact angle photographs for developed nanocomposites. Figure 4a,b depict the contact angles for DI (deionized water) and surface-free energy for NCs. The addition of NPs of SiC, WC, and ZrC increased the surface free energy (SFE), showing the transition of hydrophobic polymers to the mildly hydrophilic NCs. Table 3 depicts the D (disperse component) and P (polar component) components calculated during SFE measurements.

Contact angle (CA) has an inverse relationship with surface-free energy (SFE), while SFE has a direct relationship with lap shear strength (LSS). As seen from Figure 4a CA, these films followed the order PAEK ≈ C_WC_ < C_ZrC_ < C_SiC._ PAEK, being hydrophobic, showed the highest CA and lowest SFE. The inclusion of WC in PAEK did not affect CA or SFE appreciably. On the other hand, the inclusion of SiC led to the highest SFE followed by ZrC. In the case of LSS (Figure 4b), the selected materials followed the order PAEK ≈ C_WC_ > C_ZrC_ >C_SiC_, which is in tune with their LSS (Figure 11).

There are two surfaces and one interface in the composite: surfaces of NPs and polymers, and their interfaces. When the film interacts with the adherend, a metallic surface comes into the picture and forms the interface. The metallic surface has a higher surface energy (hydrophilic) than the ceramics and then polymers, which are hydrophobic. For good strength of the joint, the surface energy of the polymeric adhesive should be as high as possible, and hydrophilicity should be increased to the extent possible. The inclusion of ceramic NPs has increased the surface energy of nanofilms and hence their adhesion to hydrophilic metallic surfaces.

### 4.4. X-ray Diffraction Studies

**Figure 5:**
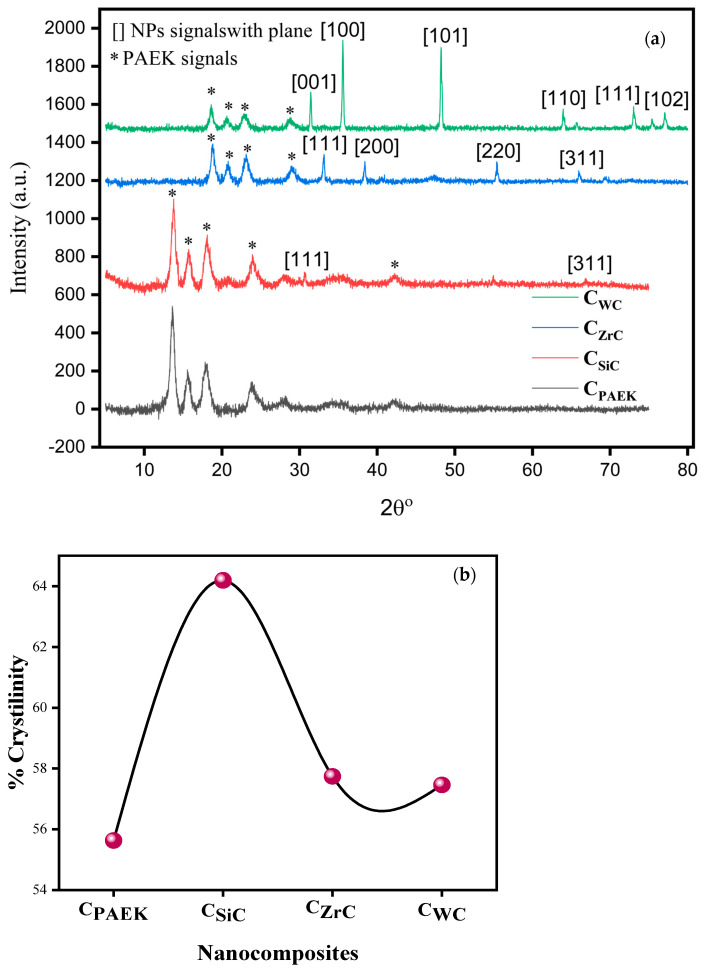
X-ray diffraction (XRD) pattern of pure C_PAEK_ and C_SiC_, C_ZrC_, and C_WC_, composites (**a**) and (**b**) shows the extent of % crystallinity.

Figure 5a depicts the X-ray diffractograms, while Figure 5b shows the extent of crystallinity in NCs. They showed a % crystallinity in the range of 50–65%, a semi-crystalline nature, and depicted nucleation-based crystallinity. However, the extent of crystallinity was found to be a qualitative function of dispersion and distribution of NPs vis-à-vis inverse function of the density of selected NPs. The higher the density of NPs, the poorer the distribution and dispersion of NPs, leading to relatively heterocrystalline NCs. The variation in the detection of the intensity of various peaks in Figure 5a depicts the indirect relation between dispersion and the density gradient formed due to heavier particles. The major diffraction peaks at 13.5°, 15.5°, 17.9°, 23.8°, 27.9°, and 42.3° are the signature peaks of pure PAEK. The gradual addition of 0.5 vol. % NPs into PAEK showed the generation of new peaks. The addition of WC into PAEK showed a signature peak at 31.3°, 35.5°, 48.01°, 64.01°, 73.1°, and 77.1° and can be indexed to [001], [100], [101], [110], [111], and [102] plains of WC, respectively. Similarly, ZrC into PAEK showed a signature at 33.0°, 38.3°, 55.4°, and 66.1° and can be indexed to [111], [200], [220], and [311] plains of ZrC, respectively. The addition of SiC NPs to PEEK also showed the new signature of SiC at 30.7° and 66.7°, which can be assigned to [111] and [311] plains. This confirms that NPs have been successfully incorporated into the PAEK. From the analysis, it can be seen that there is a 0.5 vol. % addition of SiC to the PAEK matrix.

The degree of crystallinity of the composites was calculated by finding the area of the highly intense crystalline peaks and dividing it by the total area of all the crystalline peaks in the XRD profile. The degree of crystallinity of SiC, ZrC, and WC was found to be 64%, 57.74%, and 57.46%, respectively. The peaks in C_WC_ were highly intense due to the significantly high density of WC, making them less uniformly distributed in PAEK. So, the PAEK can be easily identified in C_WC_. It has been seen that the addition of SiC NPs to PAEK alters the crystallinity of the matrix.

### 4.5. Thermogravimetric Analysis

**Figure 6:**
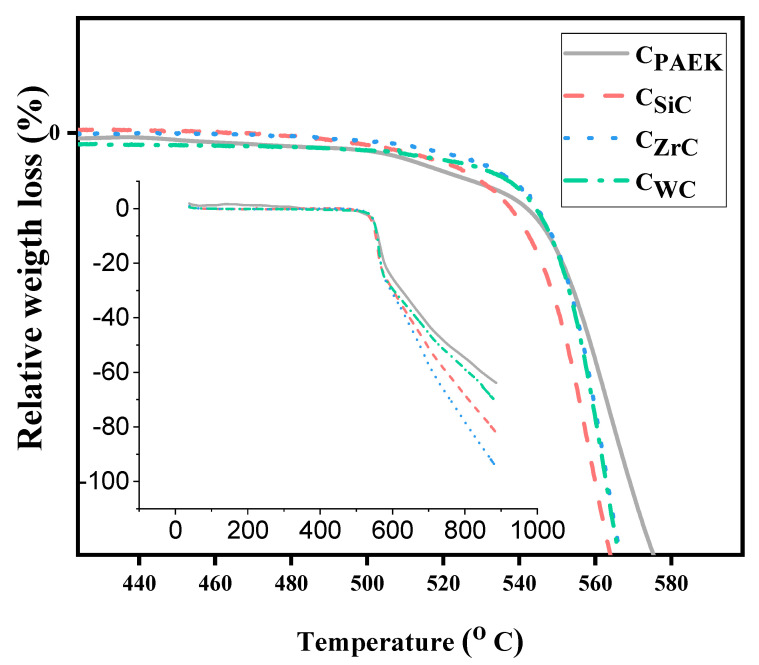
TGA thermograms for NCs.

Figure 6 depicts the change in the thermal stability of NCs with the addition of different NPs. Table 4 displays the T_5_ and T_10_ for developed NCs. The difference in thermal stability of NCs was marginal compared to the pristine PAEK, i.e., C_PAEK_. The composites’ thermal stability depends upon the added filler’s nature and thermal conductivity. It was suspected that highly thermally conductive materials act as the hotspot in the bulk of NCs, resulting in a higher degradation rate compared to relatively low thermal stability NCs, as seen in the case of C_SiC_, as the thermal conductivity of SiC is the highest amongst selected NPS. T_5_ and T_10_ support the hypothesized notion.

### 4.6. Thermal Conductivity

**Figure 7:**
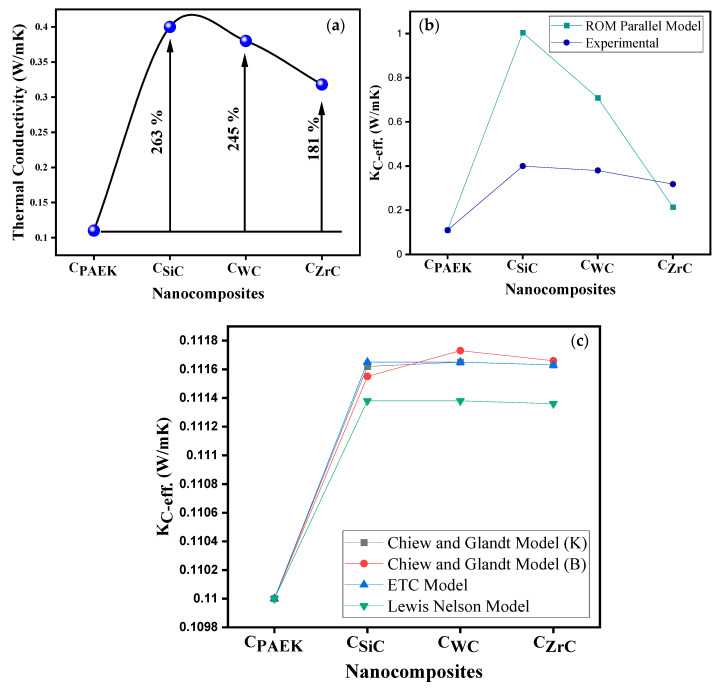
(**a**)Thermal conductivity (TC) of NCs; (**b**) theoretical models employed to predict the trend in TC and the rule of the mixture parallel model; and (**c**) Chiew and Glandt, ETC, and Lewis Nelson models.

Figure 7a depicts the thermal conductivity (TC) of NCs, which was in tune with the TC of loaded NPs.

C_SiC_ showed the highest TC, followed by C_WC_ and C_ZrC_, which depended upon the dispersion and distribution, eventually depending upon the efficiency of phonon transfer. Thus, the dispersion and distribution of NPs play a crucial role. Moreover, in the current situation, the density of selected NPs plays a crucial role in the distribution and dispersion of NPS vis-à-vis the TC of composites. Apart from it, the techniques used to prepare the nano-mixture and subsequent processing are vital due to the extent of voids they generate while processing. Generally, with higher voids, materials turn more thermally insulating than conductive due to the interruption caused by voids in the phonon transfer phenomenon.

The TC of composites agreed with the % crystallinity of the NCs (Figure 5b) due to improved phonon transfer by the ordered structure of polymeric chains (although a marginal improvement but with an extended effect). The TC showed a correlation with the LSS of the NCs (Figure 10), which might be due to the strengthening of a glue line due to the NPs induced crystallization and reinforcing effect.

Apart from experimental TC, a few theoretical models [25,26] were employed. Figure 7a–c depict the experimental results: the parallel rule of mixture (Equation (3)), Chiew and Glandt (Equation (4)), the Effective TC (ETC) model (Equation (6)), and the Lewis Nelson model (Equation (7)).

(3) $$K_{c-\mathrm{eff}}=\emptyset_{f}K_{f}+\left(1-\emptyset_{f}\right)K_{m}$$

where K_c-eff_: effective thermal conductivity of composite, Ø_f_: volume fraction of filler, K_f_: thermal conductivity of filler, and K_m_: thermal conductivity of matrix.

(4) $$K_{c-\mathrm{eff}}=\frac{1+2\emptyset_{f}\beta +\left(K-3\beta^{2}\right)\emptyset_{f}^{2}}{1-\emptyset_{f}\beta }\;$$

(5) $$\beta =\frac{\alpha -1}{\alpha +2}$$

where α = K_f_/K_m_

(6) $$\frac{K_{c-\mathrm{eff}}}{K_{m}}=\frac{1+2\beta \emptyset_{f}}{1-\beta \emptyset_{f}}$$

(7) $$\frac{K_{c-\mathrm{eff}}}{K_{m}}=\frac{1+\varepsilon \eta \emptyset_{f}}{1-\Phi \eta \emptyset_{f}}$$

η = (K_f_ − K_m_)/(K_f_ + εK_m_), Φ: 1 + ((1 − Ø_M_)/Ø_M_^2^) Ø_f_, The values of ε and Ø_M_ were taken as 1.5 and 0.637, as per the literature [25].

However, it was observed that the TC values calculated using the rule of the mixture model showed a relatively similar trend to the experimental values. The magnitude of the theoretical TC was relatively higher than the observed values. Thus, this variation could be attributed to the void content and the differential processing technique. The Chiew and Glandt model combine interaction parameters such as K and B calculated from the Nicolas-Narkis, Piggot, and Leidner models. However, the trend observed in the Chiew and Glandt and ETC Lewis Nelson models was not in tune with the experimental results and mixture rule.

### 4.7. Dynamic Mechanical Analysis

Dynamic mechanical analysis is a widely known characterization technique that is primarily used to understand the thermo-mechanical properties of materials. It resolves the viscoelastic behavior of the material over the spectrum of temperature and frequency. Apart from this, DMA could be used to understand the interaction of the reinforcing phase with the polymeric chain. The stiffening imparted by the NPs could be easily observed using DMA results.

**Figure 8:**
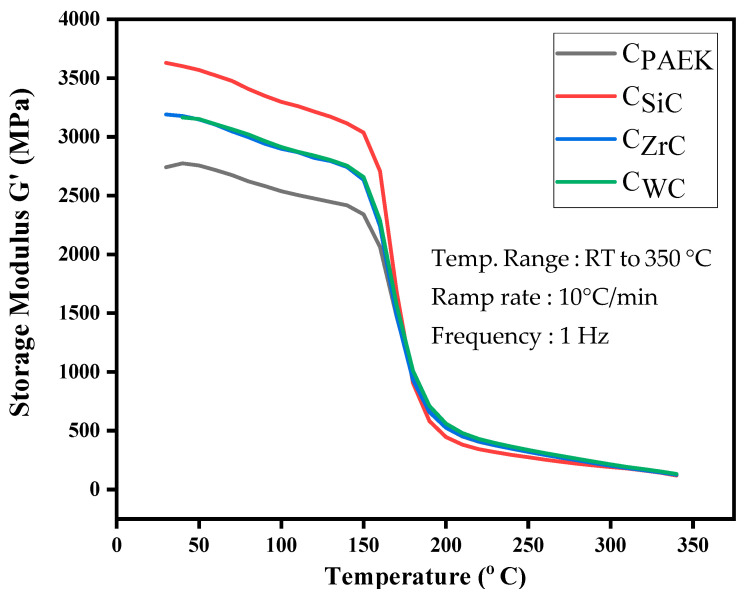
Thermomechanical stability of the NCs.

In the current study, the thermo-mechanical response of the material was recorded in tensile mode. Figure 8, which shows the storage modulus vs. temperature plot, indicates that at room temperature, the elastic phase of developed NCs dominates and deteriorates as the temperature rises. It was interesting to observe that the trend of storage modulus at room temperature matches the LSS and tensile properties. C_SiC_ with the lowest density showed the best dispersion, and DMA proved the improved interaction when the tensile load with frequency was applied. Thus, the hypothesis stating that the interaction of NPs is proportional to their density vis-à-vis efficient dispersion is a crucial aspect in the case of heavy reinforcing NPs. The rest of the NCs performed better than the pristine PAEK. Comparing C_ZrC_ and C_WC_, C_ZrC_ performed marginally better than C_WC_. Table 5 depicts the values of the storage modulus at room temperature.

### 4.8. Tensile Properties

**Figure 9:**
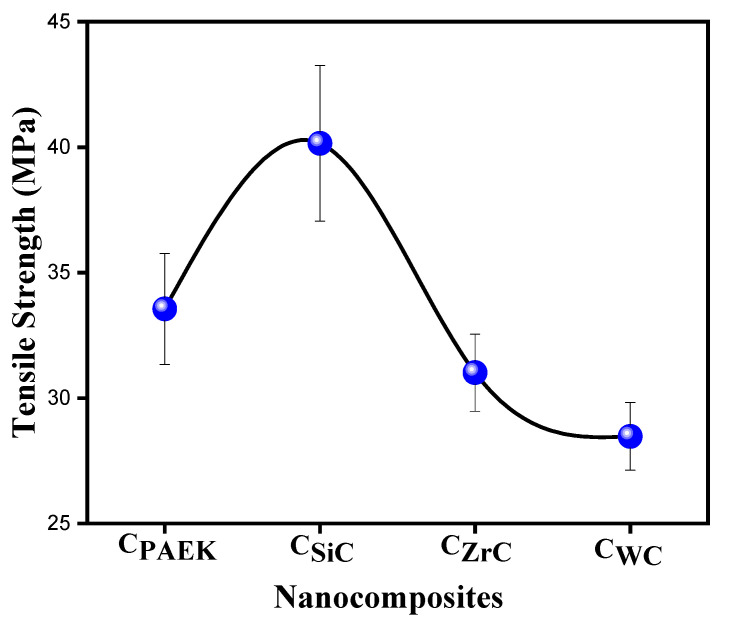
Tensile strength of developed NCs.

Figure 9 depicts the tensile strength trend for the composites. The overall lower values for the tensile strength of NCs could be due to the nature of the specimens, i.e., thin compression-molded sheets and their uneven thickness. The performance order was: C_SiC_ > C_PAEK_ > C_ZrC_ > C_WC_. The poor performance of N_WC_ could be attributed to the formation of a density gradient during the mixing of NPs due to the huge density difference (due to the high density of WC, i.e., 15.63 g/cc). One of the reasons behind the slightly better performance of SiC-based NAs might be the lower filler density. As the density of NPs increased, the inverse trend could be observed in their tensile strength.

#### The Extent of Interaction Based on Theoretical Modeling

Nicolas-Narkis and Piggot-Liedner models were used to calculate the interaction parameters for the different systems developed for various experimental studies [27,28]. The interaction parameters, such as K and B, were calculated using the tensile stress observed during the mechanical testing as per the Nicolas-Narkis and Piggot-Liedner model. Equations (8) and (9) depict mathematical relationships, respectively.

(8) $$\frac{\sigma_{c}}{\sigma_{m}}=\left(1-K\emptyset_{f}^{\frac{2}{3}}\right)$$

(9) $$\sigma_{c}={\;\sigma }_{m}\left(1-B\emptyset_{f}\right)$$

where K and B are interaction parameters, σ_c_ and σ_m_ are tensile stresses for composites and matrix, and Ø_f_ is volume fraction for fillers.

**Figure 10:**
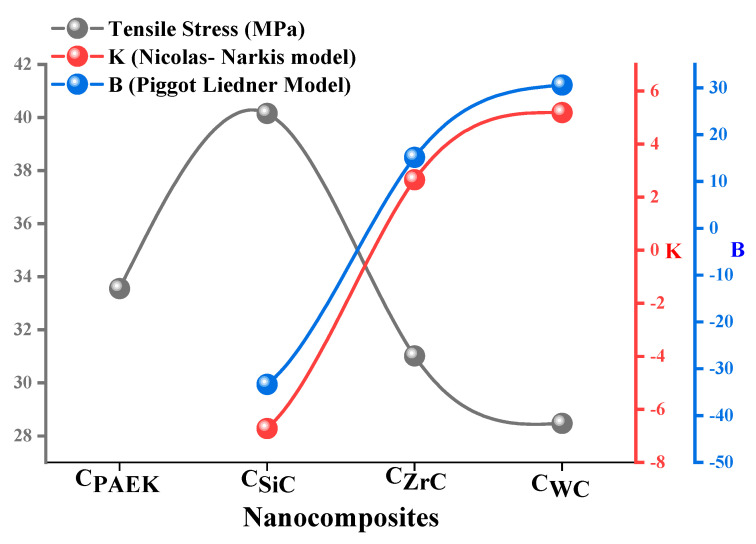
Correlation between interaction parameters and tensile stress.

The interaction parameters, i.e., K and B, provide a quantitative idea about the extent of interaction of filler particles with the matrix material [28]. As per the literature, the negative value of K and B is considered a stronger interaction. As per observed experimental trends, C_SiC_ performed well amongst the rest of the composition, reflecting its K and B values of −6.72 and −33.35 as per the given model, as shown in Figure 10. However, the values for the C_ZrC_ and C_WC_ resulted in a fragile interaction (positive values) [29]. Moreover, both models agree with the observed experimental trends, i.e., the higher the tensile strength, the lower the magnitude of the interaction parameter, and vice versa. These interaction parameters are further utilized in thermal conductivity modeling to account for the interface’s contribution to the calculation.

### 4.9. Lap Shear Strength (LSS)

**Figure 11:**
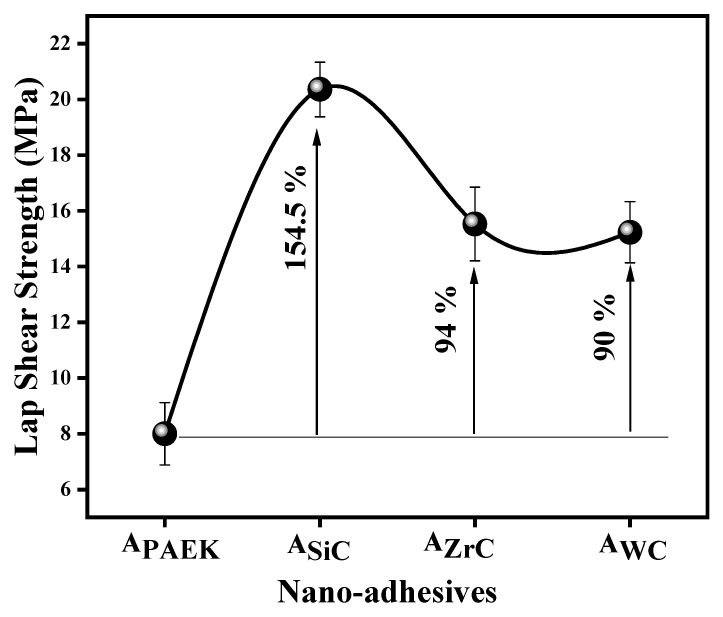
Lap shear strength of adhesives.

Figure 11 depicts the lap shear strength of nano adhesives, and the performance order is as follows:

C_SiC_ (154%) > C_ZrC_ (94%) > C_WC_ (90%) > C_PAEK_

SiC-filled nano-adhesive excelled in performance, showing a significant improvement of 154% over pristine PAEK, which could be attributed to the excellent interaction of NPs with coupon surfaces due to the highest SFE (Figure 4b). Moreover, higher hydrophilicity, i.e., higher SFE, led to higher adhesion between the metallic surfaces of coupons and embedded NPs in the matrix and hence higher friction, responsible for increasing LSS. Figure 11 and Figure 4a,b depict the correlation between LSS and contact angle, SFE.

The uniform dispersion of nanoparticles (NPs) while preparing nanosuspension is a very important step for preparing a nano-mixture after the removal of the medium. This is then used for preparing films of nanocomposites. The density of NPs affects the dispersion efficiency. The higher the density, the more difficult it is to obtain a good quality of dispersion, and hence the final film of the nanocomposite. The order of density of selected NPs was SiC (3.21 g/cc) < ZrC (6.56 g/cc < WC (15.62 g/cc), which was reflected in the quality of nanocomposite films. Thus, the better dispersion and distribution of SiC compared to other NPs led to better performance properties.

Apart from the factors mentioned earlier, the major assisting factor in strengthening NC or nano-adhesives could be NP-assisted nucleation and subsequent crystallization.

### 4.10. Failure Analysis

**Figure 12:**
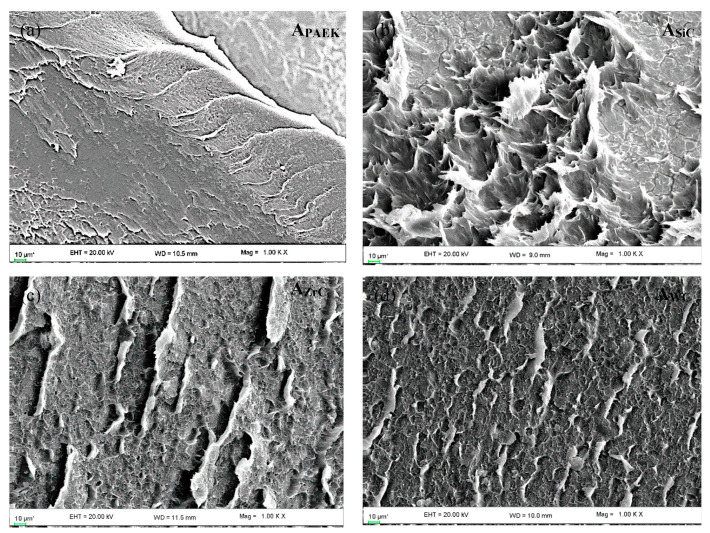
SEM mizcrographs for failed LSS joints (**a**) A_PAEK_, (**b**) A_SIC_, (**c**) A_ZrC_, and (**d**) A_WC_.

Figure 12 depicts the failure analysis of failed lap shear joints, observed with the help of SEM, to understand the dominant failure mechanism. In the case of pristine PAEK, the primary failure mechanism was mild plastic deformation resulting in adhesive failure. Adding NPs into the polymer matrix changed the failure mechanism, although all failure modes remained the same as a combination of adhesive and cohesive failure. In the case of A_SiC_, severe plastic flow with micro-striations was observed, which depicts the excellent resistance of adhesives against the applied shear force. The direction of plastic flow represents the direction of applied shear force. Similar features with smaller intensities were observed for A_WC_ and A_ZrC_; however, the pattern of plastic flow was different from A_SiC_. It could be correlated that point-to-point plastic deformation (observed for A_SiC_) is the sign of greater shear resistance followed by micro-striations and lined mild plastic deformation with micro-striation (observed for A_ZrC_ and A_WC_).

## 5. Conclusions

Using PAEK (a high-performance thermoplastic polymer) as a matrix and nanoparticles of SiC, ZrC, and WC NPs (0.5 vol. %), nanocomposites (NCs) and nano-adhesives (NAs) were developed and evaluated for various properties such as physical, thermal, thermo-physical, mechanical, thermo-mechanical, and lap shear strength.

The NPs of SiC proved more beneficial than the other two NPs. The lap shear strength also improved significantly (154%), followed by ZrC (94%), WC (90%), and PAEK. The improved LSS performance was attributed to the enhanced surface free energy of nano-adhesives due to the inclusion of ceramic NPs. Furthermore, an increase in the tensile strength of composites (in vitro studies) led to the failure of the adhesive film in bulk. These factors supported the high LSS of nano-adhesives. The failure mechanisms were mixed and shifted from adhesive failure to more cohesive failure as the contents of NPs increased. The factors affecting the performance of adhesives are as follows:

- Surface free energy;
- Tensile strength of NCs;
- Dispersion of NPs;
- Crystallinity of NCs;
- Density of NPs.

## Figures and Tables

**Table 1:** Suppliers’ data for NPs [22,23].

Properties	SiC	ZrC	WC
**Supplier**	NanoAmor (Houston, TX, USA)	Nanoshel LLC (Wilmington, NC, USA)	Nanoshel LLC (Wilmington, NC, USA)
**Purity (%)**	97.5	99.9	99.9
**Density (g/cc)**	3.21	6.56	15.62
**Particle size (nm)**	55	80–100	<100
**Surface area (m^3^/g)**	35–40	45	--
**Shape**	Nearly spherical	Nearly spherical	Nearly spherical
**Melting Point (°C)**	2730	3400	2830

**Table 2:** Designations and physical properties of the developed NCs and NAs.

NCs	NAs	Density (g/cc)	Theoretical Density (g/cc)	Composition (vol. %)	
				PAEK	NPs
C_PAEK_	A_PAEK_	1.28	1.30	100	00
C_SiC_	A_SiC_	1.32	1.30	99.50	0.5 (SiC)
C_ZrC_	A_ZrC_	1.33	1.32	99.50	0.5 (ZrC)
C_WC_	A_WC_	1.38	1.37	99.50	0.5 (WC)

C—composites and A—adhesive.

**Table 3:** SFE, contact angle (CA) (DI water, hexane), and P and D components.

	C_PAEK_	C_SiC_	C_ZrC_	C_WC_
**DI Water CA(+- error)**	90.5 (4.08)	77.3 (3.21)	84.43 (4.53)	93.77 (2.48)
**Hexane CA(+- error)**	13.52 (0.46)	12.77 (0.63)	12.82 (1.67)	15.18 (0.65)
**SFE in mN/m (+- error)**	23.12 (1.74)	32.17 (0.3)	29.83 (1.96)	22.81 (0.08)
**D comp. (+- error)**	17.89 (0.03)	17.94 (0.2)	17.95 (0.04)	17.76 (0.005)
**P comp. (+- error)**	5.23 (3.54)	14.22 (0.18)	11.89 (1.92)	5.04 (0.03)

**Table 4:** T_5_ and T_10_ for developed composites derived from TGA thermograms.

Nanocomposites	C_PAEK_	C_SiC_	C_ZrC_	C_WC_
T_5_ (°C)	547.9	549.3	554.6	554.6
T_10_ (°C)	559.4	558.9	562.5	562.7

**Table 5:** Storage modulus of developed NCs.

NCs	C_PAEK_	C_SiC_	C_WC_	C_ZrC_
**Storage modulus (MPa)**	2742	3630	3171	3191

## Data Availability

There is no data to report.
